# Epoxidized Jatropha Oil as a Sustainable Plasticizer to Poly(lactic Acid)

**DOI:** 10.3390/polym9060204

**Published:** 2017-06-02

**Authors:** Buong Woei Chieng, Nor Azowa Ibrahim, Yoon Yee Then, Yuet Ying Loo

**Affiliations:** 1 Department of Chemistry, Faculty of Science, Universiti Putra Malaysia, 43400 UPM Serdang, Selangor, Malaysia; norazowa@upm.edu.my; 2 Materials Processing and Technology Laboratory, Institute of Advanced Technology, Universiti Putra Malaysia, 43400 UPM Serdang, Selangor, Malaysia; 3 Department of Pharmaceutical Chemistry, School of Pharmacy, International Medical University, No. 126, Jalan Jalil Perkasa 19, Bukit Jalil, 57000 Kuala Lumpur, Malaysia; yoonyeetyy84@yahoo.com; 4 Department of Food Science, Faculty of Food Science and Technology, Universiti Putra Malaysia, 43400 UPM Serdang, Selangor, Malaysia; yuet_ying88@hotmail.com

**Keywords:** jatropha oil, epoxidized oil, plasticizer, poly(lactic acid)

## Abstract

A renewable resource, epoxidized jatropha oil (EJO), was used as a green plasticizer and added to poly(lactic acid) (PLA). EJO was compounded into PLA at different contents. The addition of 3 wt % EJO to the PLA demonstrates significant improvement in flexibility, which leads to a percentage increase of about 7000% in elongation at break. This tensile result was confirmed by surface morphology analysis with clear proof of plastic deformation in EJO-plasticized PLA. EJO imparts a good heat stabilization effect. Thermal stability of PLA was enhanced upon addition of EJO, which is due to their good interaction and plasticizer dispersion within the PLA matrix. This EJO-plasticized PLA has wide applications in various industries, such as packaging of food and non-food products.

## 1. Introduction

In recent years, there has been a growing environmental concern regarding the use of polyolefin-based plastics such as poly(ethylene) (PE), poly(propylene) (PP), and poly(ethylene terephthalate) (PET), which are non-biodegradable. This non-biodegradable nature of such products results in plastics pollution, which involves a rapid buildup of plastic waste in landfills and increases in greenhouse gas emission. All of these problems related to the use of non-biodegradable polymer or synthetic plastics have urged scientists to search for new materials that are bio-inspired or bio-based as an alternative. Biodegradable plastics or biopolymers offer a solution and are receiving a significant amount of attention because they are environmentally friendly, biodegradable, compostable, and derived from renewable natural and sustainable biomass resources. Moreover, they decrease global dependency on depleting fossil fuels and reduce the generation of hazardous substance.

Poly(lactic acid) (PLA), a biodegradable polymer has been proposed as a solution to the waste problems related to non-biodegradable polymers. PLA-based materials have many advantages over their non-biodegradable competitors, such as stiffness, transparency, and recyclability. Unfortunately, the brittleness and poor barrier properties are the main challenges restricting their use and competing with conventional plastics, particularly in the field of flexible films. Therefore, to extend the range of PLA applications, considerable efforts have been made to enhance its properties by employing various plasticizers such as poly(ethylene glycol) (PEG) [1,2], oligomeric lactic acid (OLA) [3], tributyl citrate (TBC) [4], and octyl epoxy stearate (OES) [5]. Another interesting approach is the use of environmentally friendly and non-toxic vegetable oil-based plasticizer.

Vegetable oils are chemically composed of glycerols and fatty acids. As vegetable oil is derived from plants, it is biodegradable, renewable, and relatively inexpensive for various industrial applications. Edible vegetable oils, such as palm oil, soybean oil, rapeseed oil, and sunflower oil, have all become major sources for plasticizer production in recent decades. Extensive research on epoxidized palm oil as a plasticizer has been conducted by researchers from Malaysia, including Al-Mulla et al. [6], Silverajah et al. [7], Tee et al. [8], and Chieng et al. [9]. In Malaysia, oil palm plantations possess higher productivity, with the lowest oil production cost as compared to other oil-producing crops [10]. This keeps the price of palm-based plasticizer competitive enough to meet the demand of the commodity market. However, there are serious concerns regarding the use of these edible oils as plasticizer feedstock. This edible plasticizer feedstock may compete with food supply and cause the edible oil price increase in the near future. Environmental issues such as deforestation will likely develop as the massive propagation of plants producing edible oil takes place. As an alternative, researchers have focused on non-edible oil for plasticizer production to overcome these drawbacks.

Amongst the varieties of non-edible vegetable oil, jatropha oil (JO) is the most favorable for plasticizer production due to the relatively high content of unsaturated fatty acids. The jatropha plant, *Jatropha curcas*, belongs to the Euphorbiaceae family. The jatropha plant is a multipurpose, drought-resistant, oil-bearing plant, originating from South America [11]. The kernel seed of the jatropha plant contains the most valuable oil, consisting of 40% by weight of triacylglycerol with linear fatty acid chains. This high oil content indicated that the jatropha plant is suitable as a feedstock for plasticizer production. JO is not suitable for daily nutrient consumption purposes due to the presence of toxic compounds in oil such as curcin and purgative, but it has many medicinal values. Thus, JO is non-edible vegetable oil and traditionally used for soap manufacturing and medicinal applications [12].

In general, the use of plasticizer as a modifier to PLA is bound by its safety, environmental favorability, and chemical and physical properties, which dictate its miscibility and required flexibility towards the target applications. The plasticizer leaching during storage or end-user applications, as well as toxicity, has become a major safety risk and restricts plasticizer from applications in many industries, including medical, pharmaceutical, and food packaging fields. The ideal plasticizer originates from renewable sources, are biodegradable, stable, and toxic-free, and exhibit no or minimum leaching or migration during use or aging. Therefore, it is advantageous to provide a plant-based, non-edible oil of *Jatropha curcas* for use as a plasticizer in the PLA biodegradable polymer, which is capable of overcoming at least one of the above-mentioned problems. In this study, we report our findings on the application of the epoxidized jatropha oil (EJO) as a plasticizer to improve the flexibility of the PLA polymer.

## 2. Materials and Methods

### 2.1. Materials

Commercial grade Poly(lactic acid) 4042D resin with an *M*_W_ of ~390,000 g/mol, was supplied by NatureWorks^®^ LCC, Minnetonka Blvd, MN, USA. Crude jatropha oil (JO) was purchased from Bionas Sdn Bhd, Kuala Lumpur, Malaysia.

### 2.2. Epoxidation of Jatropha Oil

The EJO was synthesized by in situ epoxidation with molar ratio of 1:0.6:1.7 (JO double bond: formic acid/hydrogen peroxide). Firstly, 10 g of formic acid was added to 100 g of JO in a reaction flask. The reaction flask was kept stirring at a speed of 250 rpm in a water bath at 40 °C. At the same time, 135 mL of hydrogen peroxide (H_2_O_2_) was added slowly to the reaction mixture over a time range of 30 min in order to prevent the solution from overheating. After 30 min, the temperature increased to 60 °C, and the solution was stirred continuously for another 4 h. The EJO was then cooled down to room temperature and washed with distilled water until it was neutral. The characteristics of the EJO obtained are listed in Table 1.

### 2.3. Preparation of EJO-Plasticized PLA

The EJO-plasticized PLAs were prepared by melt blending technique using Brabender Internal Mixer (Duisburg, Germany) with a mixing speed of 50 rpm and a temperature of 170 °C for 10 min [13]. PLA resin was first dried in the oven for 24 h to prevent potential hydrolytic degradation prior to the mixing process. PLA resin was melted in a mixing chamber for 3 min before the EJO plasticizer was added. The weight of EJO studied varied from 0 to 10 wt %. The compounded materials obtained were then molded and compressed into sheet form with a thickness of 1 mm by hot-pressing at 165 °C for 10 min with a pressure of 110 kg/cm^2^, followed by cold-pressing at 30 °C for 5 min. The final products in sheet form were used for the following characterizations. 

### 2.4. Characterizations

#### 2.4.1. Fourier-Transform Infrared (FTIR)

FTIR spectra were recorded using a Perkin Elmer Spectrometer Model 1000 Series (Waltham, MA, USA) equipped with a Universal Attenuated Total Reflectance (UATR). The spectra were recorded from a frequency of 280 to 4000 cm^−1^. The FTIR Spectrum Software (Perkin Elmer) program was used to analyze the data. 

#### 2.4.2. Tensile Properties

A tensile properties test was performed using Universal Testing Machine Instron (Buckinghamshire, UK) Model 4302 series IX. The 1 mm EJO-plasticized PLA sheets were cut into a dumbbell shape following the ASTM D638 (type V) standard. The test was conducted at room temperature with a 1.0 kN load cell and a constant crosshead speed of 10 mm/min. A tensile strength, tensile modulus, and elongation at break were evaluated from the stress–strain data. Result of five tested replicates for each formulation was reported to obtain a reliable average value and standard deviation.

#### 2.4.3. Thermogravimetric Analysis (TGA)

Thermal analysis of EJO-plasticized PLA was carried out using a Perkin Elmer Model Pyris 7 (Waltham, MA, USA) analyzer with a scan range from 35° to 800° at a constant heating rate of 10 °C/min and continuous nitrogen flow of 20 mL/min. Temperatures at onset (*T*_onset_), maximum weight loss (*T*_max_) and 50% weight loss (*T*_50_) were determined from the thermogram obtained.

#### 2.4.4. Differential Scanning Calorimetry (DSC)

DSC analysis was performed on a Mettler Toledo model DSC 822e (Columbus, OH, USA). The samples were first heated from 30 to 180 °C with a heating rate of 10 °C/min, they were then held at this temperature for 5 min to eliminate the thermal history, and they were then cooled to 30 °C at a cooling rate of 10 °C/min and held at 30 °C for 5 min. Finally, they were reheated to 180 °C at a heating rate of 10 °C/min.

#### 2.4.5. Morphology

The fractured sample from the tensile test was collected and sputter-coated with a thin layer of gold to prevent a charging effect prior to surface observation. The fracture surfaces were observed under a JEOL SEM Model JSM-6400 (Tokyo, Japan) at an accelerating voltage of 15 kV. 

## 3. Results and Discussion

**Figure 1:**
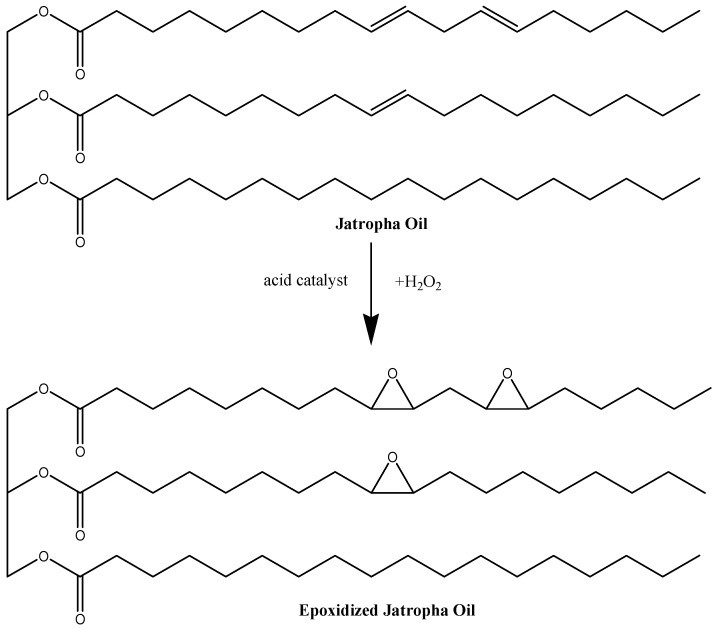
Epoxidation reaction of jatropha oil (JO).

Epoxidaton reaction, which adds an oxygen atom to a carbon–carbon double bond, has been established as an important method for the formation of carbon–oxygen bonds [14]. Unsaturated fatty acids were the components of interest in the epoxidation of vegetable oils due to the presence of carbon–carbon double bonds required in the reaction. JO contains high unsaturated fatty acid content, including oleic (C18:1) and linolenic (C18:2) acid, and was suitably converted to an epoxide group via epoxidation to produce epoxidized jatropha oil (EJO). Daniel et al. [15] reported that the average number of double bonds in a fatty acid of JO is about 1.08–1.13, the fraction of unsaturated fatty acid is 0.78–0.79, and the average carbon length of fatty acid chain is 17.3–17.8. Figure 1 shows the structure and epoxidation process of JO to form EJO.

**Figure 2:**
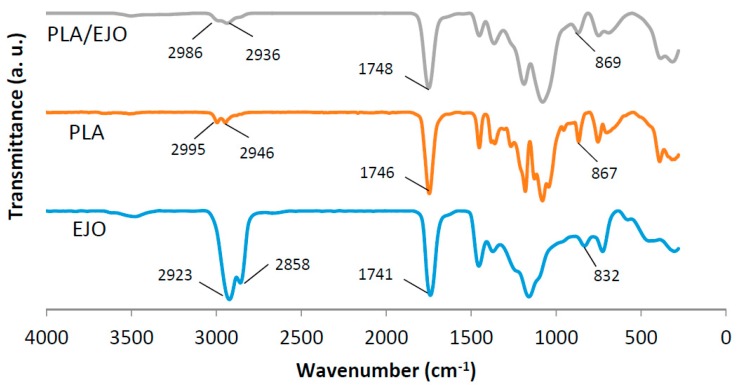
FTIR spectra of poly(lactic acid) (PLA), EJO, and EJO-plasticized PLA.

Figure 2 shows the FTIR spectra of PLA, EJO, and EJO-plasticized PLA. Typical characteristic peaks of PLA were stretching vibrations of –CH_2_ (2995 cm^−1^ and 2964 cm^−1^) and –C=O (1746 cm^−1^). EJO reveals the aliphatic –C=O stretching of ester at a wavelength of 1741 cm^−1^. In addition, two strong peaks at around 2900–3000 cm^−1^ were derived from the –CH_2_ stretching vibrations. The presence of the epoxide group in EJO was proven by the stretching band at 832 cm^−1^. The disappearance of this epoxide stretching vibration peak in EJO-plasticized PLA indicated the possibility of interaction between the EJO plasticizer and PLA. This finding is similar to PLA/epoxidized palm oil (EPO) and PLA/epoxidized soybean oil (ESO) reported by Silverajah et al. [16] and Tee et al. [17], respectively. Silverajah et al. proposed that the possible interaction was raised from the hydrogen bonding between the terminal hydroxyl group of PLA and the epoxide group of EPO.

The interaction between the polymer and plasticizer (PLA-EVO) contributes to hydrogen bonding, which is influenced by the epoxy content, also known as the oxirane oxygen content (OOC), of the epoxidized oils. The OOC value indicates the epoxy groups present in the plasticizer. A higher OCC value in EPSO (3.58%) compared to EPO (3.23%) resembles stronger interaction (hydrogen bonding) between PLA and EPSO, which gives better tensile properties.

**Figure 3:**
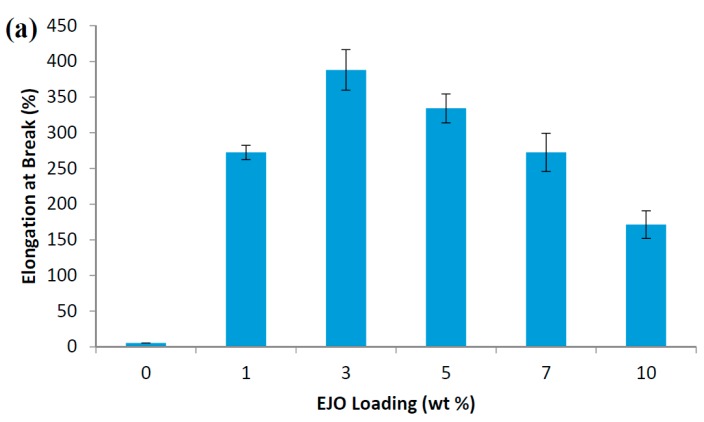

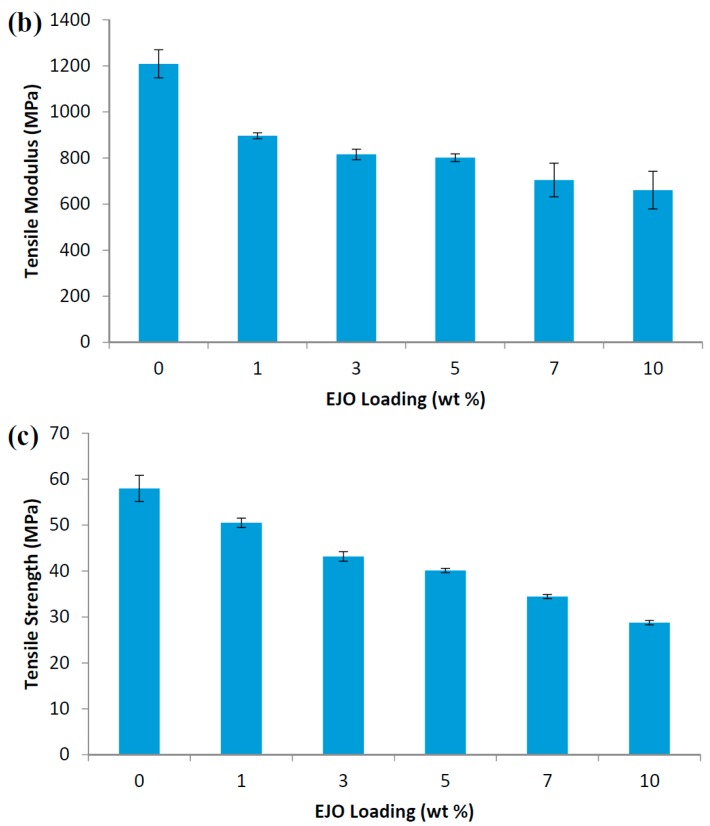
Tensile properties of EJO-plasticized PLA: (**a**) elongation at break; (**b**) tensile modulus; (**c**) tensile strength.

The tensile results of EJO-plasticized PLA are shown in Figure 3. The pristine PLA is typically rigid and brittle in nature. PLA has a very low flexibility or elongation at break of 5.37%, despite the fact that it has very high tensile strength and tensile modulus. The elongation at break of PLA was significantly increased after being plasticized by EJO plasticizer. For example, the elongation attained the highest value of 388.03% when 3 wt % EJO plasticizer was incorporated into the PLA matrix. It shows around 7000% improvement compared to pristine PLA. However, a higher amount of EJO content led to a decrease in elongation at break because PLA was saturated with plasticizer and phase separation occurred, leading to the formation of PLA-rich and EJO-rich phases within the EJO-plasticized PLA. Interestingly, the elongation at break improvement was very pronounced compared to epoxidized palm oil (EPO) and epoxidized soybean oil (ESO). Silverajah et al. [16] reported an improvement of around 1500% in the elongation at break for EPO-plasticized PLA (tested at a crosshead speed 5 mm/min, dumbbell specimen), while Tee et al. [17] reported even lower increments of around 900% (tested at a crosshead speed of 5 mm/min, rectangular specimen), both at 3 wt % EPO. For the ESO plasticization effect on PLA, both Tee et al. and Xu et al. [18] revealed that there was no significant increase in ESO-plasticized PLA at ESO loading of 3 wt %.

**Figure 4:**
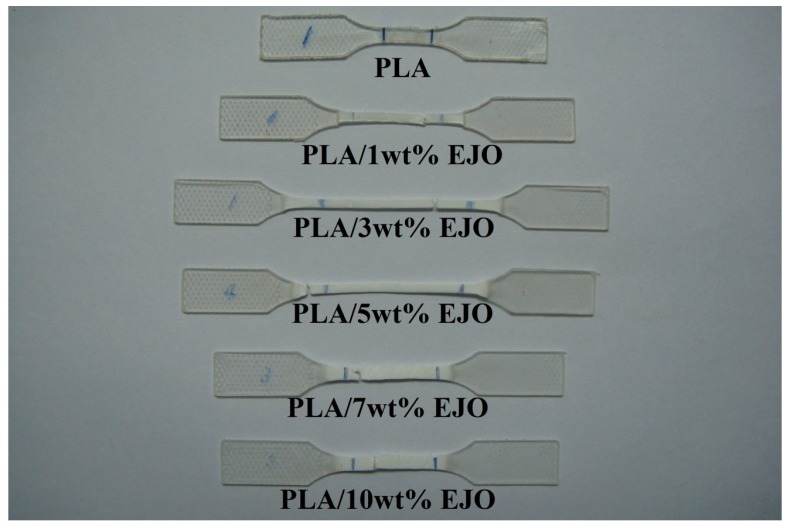
Picture of EJO-plasticized PLA after the tensile test.

On the other hand, the tensile modulus of EJO-plasticized PLA decreases at 1 wt % EJO, it remains stable at 3% and 5% EJO, and a second slight drop occurs at 7 and 10% EJO (Figure 3b). A gradually decrease in tensile strength of EJO-plasticized PLA from 57.98 to 28.76 MPa was observed as the content of EJO increased from 0 to 10 wt % (Figure 3c). An increase in the elongation at break means that the brittleness of samples decreases since the elongation at break and brittleness are inversely proportional. The change in the flexibility of PLA upon the addition of EJO can be observed in Figure 4, where the tensile deformed samples exhibited stress whitening and appeared to be more elongated in length compared to the rigid PLA.

**Figure 5:**
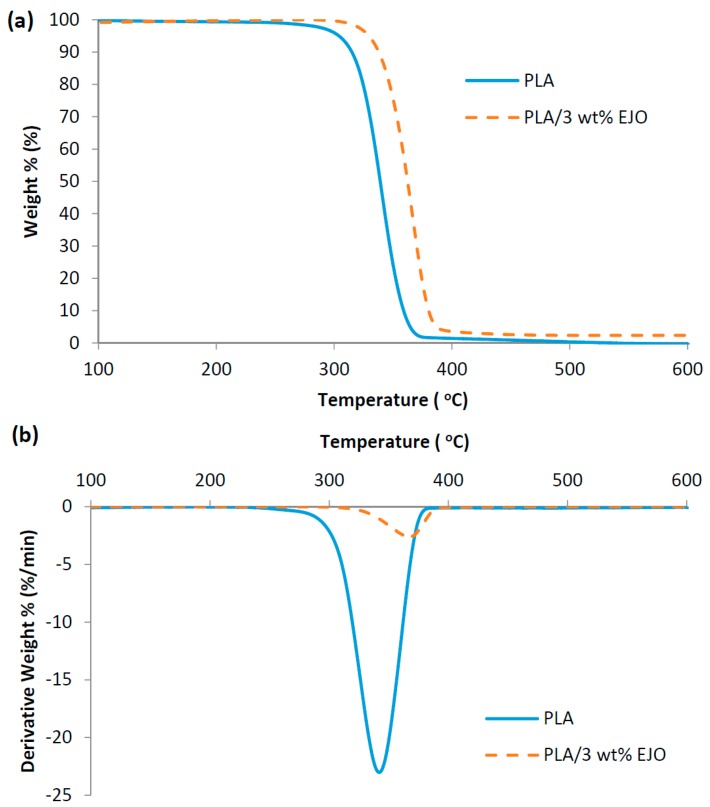
(**a**) TG; and (**b**) DTG thermograms of PLA and PLA/3 wt % EJO.

The thermal characteristics of PLA and EJO-plasticized PLA were investigated by means of TGA and DSC. The thermogravimetric curves from TGA provide information about the nature and extent of degradation of the polymeric materials. Figure 5 shows the (a) TG and (b) DTG of PLA and EJO-plasticized PLA. The thermogravimetric behavior of EJO-plasticized PLA, similar to PLA, revealed only one major degradation step at around 300–400 °C.

Thermal characteristic factors such as initial decomposition temperature (*T*_onset_), temperature of maximum rate of degradation (*T*_max_), and decomposition temperature at 50% weight loss (*T*_50_) can be determined from the TG and DTG thermograms, and the results are tabulated in Table 2. Neat PLA has a *T*_onset_, *T*_max_, and *T*_50_ of 274.26, 345.12 and 339.16 °C, respectively. The addition of 3 wt % EJO into PLA improved the thermal stability of PLA, as can be seen from the increased *T*_onset_ (303.17 °C), *T*_max_ (362.81 °C), and *T*_50_ (362.33 °C), compared to those of neat PLA. It was reported in the literature that an increase in thermal stability of epoxidized oil-plasticized PLA was due to their good interaction and plasticizer dispersion within the PLA matrix [3,9,19]. Furthermore, the homogeneously and well dispersed EJO could act as a protective layer, which deterred the release of volatile degradation products out from the composites and therefore delayed the thermal degradation [7]. 

**Figure 6:**
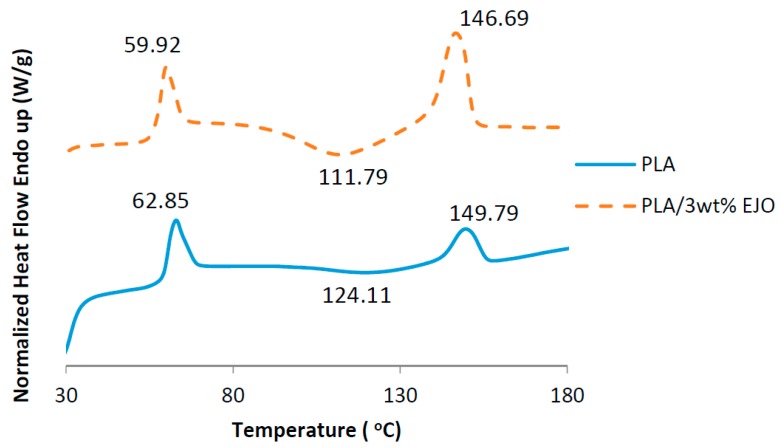
DSC thermograms of PLA and EJO-plasticized PLA.

The DSC thermograms of PLA and EJO-plasticized PLA are shown in Figure 6. The PLA shows a sharp glass transition temperature (*T*_g_) at 62.85 °C, and a melting temperature (*T*_m_) at 149.79 °C, but no obvious crystallization exotherm (*T*_c_) peak was observed. The addition of EJO plasticizer to PLA induces a shift of *T*_g_ to a lower temperature, e.g., from 62.85 to 59.92 °C, which is due to an enhanced chain mobility of PLA. Enhanced PLA chain mobility further promotes *T*_c_ and thus the *T*_m_ of PLA at around 111.79 and 146.69 °C, respectively, as can be seen in EJO-plasticized PLA thermogram. This is typical plasticized thermoplastics behavior as reported by many researchers [20].

**Figure 7:**
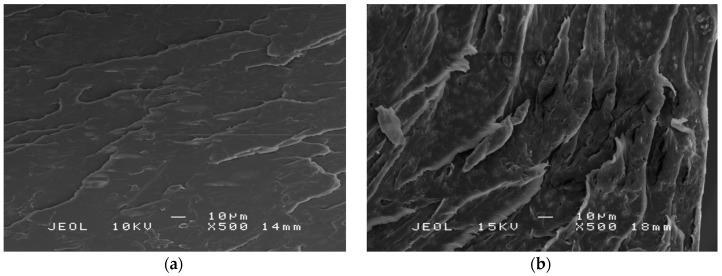
SEM micrographs of (**a**) PLA and (**b**) PLA/3 wt % EJO.

SEM was employed to examine the surface morphology of fractured tensile samples and the state of EJO dispersion in the PLA polymer matrix. Figure 7a shows a fracture surface of PLA, which exhibited flat and smooth surface corresponding to brittle crack growth behavior. Meanwhile, the fracture surface of EJO-plasticized PLA shows a typical ductile material characterized by an uneven surface and the presence of fibrils due to plastic stretching or deformation, as shown in Figure 7b. No microvoids of EJO were observed in EJO-plasticized PLA, which signified that EJO was homogenously mixed with the PLA matrix and good miscibility. The incorporation of EJO as a plasticizer into the PLA matrix determined remarkable changes on the morphology due to the enhanced interfacial adhesion and EJO dispersion. PLA changed the tough and brittle nature of the flexible materials, which is consistent with the tensile test results discussed in the previous section.

## 4. Conclusions

This study presents the potential of non-edible EJO as a plasticizer for PLA that can replace the current dependence on edible oil resources. PLA was plasticized with green EJO plasticizer at different loadings. The use of EJO plasticizer as a modifier of PLA meets multiple criteria, such as biodegradability and non-toxicity. EJO plasticizers could constitute attractive alternatives for petroleum-based plasticizers such as phthalates. The addition of EJO to PLA demonstrates remarkable improvements in flexibility. Three weight percent EJO leads to a percentage increase in elongation at break of about 7000%. This behavior was confirmed by surface analysis of fractured sample by SEM with clear evidence of plastic deformation in EJO-plasticized PLA samples. EJO-plasticized PLA has great potential as an alternative to widely used polyolefin-based flexible plastics such as PP and PE. Nevertheless, the EJO plasticizer leaching during storage and toxicity remain unknown. The ideal plasticizer should originate from renewable sources, be biodegradable, stable, and toxic-free, and exhibit no or minimum leaching or migration during use or aging. Therefore, future investigations are needed to understand the migration possibility or the migration mechanism of EJO-plasticized PLA.

## Figures and Tables

**Table 1:** Characteristics of epoxidized jatropha oil (EJO).

Characteristic	Epoxidized jatropha oil
Oxygen Oxirance Content (%)	4.30
Acid Value (mg·KOH/g)	4.63
Iodine Value (g·I_2_/100 g)	0.68
Moisture Content (%)	0.08
pH	5–6

**Table 2:** Summary of thermal degradation temperatures of PLA and PLA/3 wt % EJO.

	*T*_onset_ (°C)	*T*_max_ (°C)	*T*_50_ (°C)
PLA	274.26	345.12	339.16
PLA/3 wt % EJO	303.17	362.81	362.33
